# Exploring contextual demands that challenge nursing students in their self-directed learning of bioscience: a comparative qualitative content analysis

**DOI:** 10.1186/s12909-026-08840-5

**Published:** 2026-02-21

**Authors:** Monika Ravik, Hanne Nørbygaard Bjerva, Charlotte Weider, Karin Bölenius

**Affiliations:** 1 University of South-Eastern Norway, Borre, Norway; 2 Department of Nursing, Umeå University, Umeå, Sweden

**Keywords:** Anatomy, Bachelor nursing programme, Biochemistry, Biosciences, Busy lives, Nursing students, Physiology, Self-directed learning

## Abstract

**Background:**

Nursing students often find self-directed learning in anatomy, physiology, and biochemistry challenging to manage alongside busy lifestyles. To address these challenges, it is essential for nurse educators to understand the specific contextual demands that hinder students’ self-directed learning. This study aims to explore and compare contextual demands that challenge young and mature nursing students’ engagement in efficient self-directed learning in bioscience.

**Methods:**

A comparative qualitative content analysis study was conducted to explore contextual demands. Data were collected in November 2024 through written submissions from 98 nursing students. These submissions were analysed via qualitative content analysis to identify and compare patterns related to the contextual demands that challenged self-directed learning between two student groups. This approach provided a clear overview of key patterns, highlighting similarities and differences in the distribution of contextual demands challenging self-directed learning in anatomy, physiology, and biochemistry.

**Results:**

The analysis revealed five major categories of challenges: ‘work-related conditions’, such as part-time jobs; ‘social-related conditions’, including social commitments and family responsibilities; ‘conditions related to physical activity’, where students had to balance exercise and academic demands; ‘conditions related to social media usage’, highlighting how digital distractions impacted study focus; and ‘long commutes between home and the academic institution’, which reduced the time available for learning.

**Conclusions:**

The results emphasize the distribution of contextual demands that challenge self-directed learning in biosciences in nursing education, and highlight the need for detailed strategies to address these aspects. The results pave the way for future research and potential policy adjustments to better support nursing students in managing their academic and personal obligations.

**Supplementary Information:**

The online version contains supplementary material available at 10.1186/s12909-026-08840-5.

## Contribution of the paper

### What is already known

- Knowledge in anatomy, physiology, and biochemistry is crucial for nurses to recognize symptoms, anticipate complications, and deliver appropriate care.
- Nursing students struggle with anatomy, physiology, and biochemistry due to the volume and complexity of material and the extensive self-directed learning demands.
- There is a need for greater insight into self-directed learning among nursing students in the areas of anatomy, physiology, and biochemistry.

### What this paper adds

- Both younger and mature nursing students struggled to balance self-directed learning in biosciences with heavy workloads.
- Younger nursing students faced distractions from technology, social media, and the need to maintain a social life, whilst mature students dealt with parental responsibilities.
- Physical activity was a time-consuming but essential factor for younger students’ well-being, whereas mature students did not view it as a distraction.
- Lengthy commutes negatively impacted SDL opportunities for both younger and mature student groups, highlighting the need for targeted educational strategies.

## Background

A central part of the initial bachelor’s degree (BA) nursing programme is the study of bioscience, including anatomy, physiology, and biochemistry [1,2]. Bioscience knowledge is essential for developing critical nursing skills, such as understanding the patient’s condition better, interpreting potential complications, and administering appropriate care [3, 4]. However, many nursing students find bioscience challenging to learn, with a significant number failing exams during the initial phase of the BA nursing programme [1, 5–8]. This high rate of failure in bioscience exams is also documented in allied health sciences courses [9, 10], and is therefore a challenge for more than just students in the nursing programme. These challenges stem from the large volume of material, the complexity of integrating knowledge across biological systems, inadequate teaching strategies, and the limited time available for students [4, 8, 11–13]. In the study by Horiuchi-Hirose et al. [4], many students found anatomy and physiology challenging to learn due to the lack of connection to practical situations. Registered nurses (RNs) are not always responsible for teaching biosciences, which makes it challenging to link the subject to nursing-related subjects [1]. Even the use of technology that students perceive as effective learning tools does not necessarily provide them with appropriate learning outcomes in biosciences [14]. Importantly, university education seeks to cultivate students’ independence and self-management skills, which are critical for future professional challenges in the health sector [15, 16]. However, despite these efforts, many students struggle to keep up with the demands of extensive self-directed learning (SDL) that are required to master the complex subject of bioscience [8, 13, 14].

SDL involves students taking the initiative in diagnosing their learning needs, setting learning goals, identifying appropriate learning resources, and evaluating their learning outcomes at their own pace. In the context of bioscience, SDL requires a reflective and deep learning approach, which is often linked to metacognitive strategies that encourage critical thinking and self-awareness [14, 15, 17]. This contrasts with a surface learning approach, which tends to focus on rote memorization rather than fostering a comprehensive understanding of the material [18]. Although this learning approach fosters independence, critical thinking, and problem-solving skills, many students struggle to balance SDL with other academic and personal responsibilities [17, 19]. Nursing students often juggle part-time jobs, family obligations, and other commitments, which limits the time and energy that they can dedicate to SDL [20, 21]. The challenge of managing these competing responsibilities, combined with the rigorous workload of academic studies, is further compounded for both younger and mature nursing students [22]. Added to this, the unpredictability caused by the COVID-19 pandemic and the need for rapid adaptation likely shifted students’ focus towards immediate coping strategies rather than long-term planning and problem-solving [19]. However, challenges with learning were also a pre-pandemic concern, as for first-year bioscience students, self-directed learning appeared to be driven more by assessment requirements than by a genuine motivation for deep learning [12]. As a result, this constant balancing act leaves students with limited opportunities to fully engage with and internalise complex learning objectives, which ultimately hinders their ability to develop a deep understanding of the material [22, 23].

A detailed analysis of mature nursing students and their SDL is lacking [7, 24], and younger nursing students may find SDL particularly challenging due to a lack of prior experience in managing their own education [1, 19, 25]. Many younger students transition directly from high school, where learning environments are typically structured, teacher-led, and heavily supervised [26]. The shift to higher education, which requires greater independence and self-management, can therefore feel overwhelming for novice nursing students [19, 26]. Future research should focus on gaining a deeper understanding of SDL in nursing education, with an emphasis on identifying the unique challenges for engaging in SDL that are faced by students of different age groups [6, 7, 27–30]. Tailored interventions are needed to equip students with the skills to effectively manage SDL, addressing the specific demands of their academic and personal lives [19, 31, 32]. By optimizing learning strategies for both young and mature nursing students, educators can ensure that students acquire the competencies that are necessary for SDL and success in their studies and future careers.

The present study therefore aimed to explore and compare the contextual demands that challenge young and mature nursing students’ engagement in efficient SDL of biosciences.

## Methods

### Study design

This study employed a comparative qualitative content analysis design, to systematically explore and compare the specific contextual demands that contribute to SDL challenges among nursing students. Qualitative content analysis is a systematic research method that is used to quantify and analyse the content and relationships of certain words, themes/categories, or concepts within qualitative data [33, 34]. A comparative research design was implemented, involving the analysis of numerical data to compare and describe the nature of the phenomenon between two student nurse groups – younger and mature students [35]. Younger and mature students may have different learning needs and preferences [36, 37].

### Nursing education context

To qualify as registered nurses in Norway, nursing students must successfully complete a three-year bachelor’s degree programme, which is equivalent to 180 ECTS (European Credit Transfer and Accumulation Transfer System) and includes academic, simulation-based, and clinical placement learning [38, 39]. During their first year of education, the nursing students study biosciences and are required to take exams with these subjects at the end of the first semester.

For students in the current study, the course in anatomy, physiology, and biochemistry was delivered alongside other demanding subjects, such as microbiology, nursing science, and simulation-based practical skills learning. This parallel structure required students to manage multiple academic learning outcomes simultaneously, thereby further intensifying the complexity of their learning experience.

### Study participants and recruitment

The participants were first-year nursing students in a bachelor’s programme in nursing at one university campus in southern Norway. Following approval from the Vice-Dean of the Faculty of Health Sciences, all 147 first-year nursing students were invited to participate in the study. The inclusion criterion was students in their first semester of the nursing programme. Students who were retaking the bioscience exam because they had not passed the previous exam were excluded from the study. In a classroom setting where students preparing for their first exam were gathered, they were all provided with the same comprehensive information about the study. This included details about its aims, procedures, and ethical considerations. They were also given a statement of consent to review and sign. In the end, 98 students consented to participate in the study. The young student group comprised 69 students, with a mean age of 20.7 years, whereas the mature student group consisted of 29 students, with a mean age of 34.3 years. The term mature student typically refers to an individual who returns to education later in life, often after a period away from formal education. For the purposes of this study, owing to the ambiguity surrounding the definition of a mature student [27], we define a mature student as someone who is 26 years of age or older.

### Data collection

The empirical material for this study consisted of written text narratives, with each student providing one submission of approximately 150 words, which were collected in November 2024. These narratives were submitted to the educational institution’s digital learning platform and addressed the contextual demands that contribute to the SDL challenges of biosciences (anatomy, physiology, and chemistry). The written narratives were based on the following question: What factors prevent you from dedicating sufficient time to SDL?

The detailed and context-specific insights from each student’s text narrative ensured information power, safeguarding a robust understanding of the contextual demand challenges regarding SDL that were encountered by the nursing students [40].

#### Data analysis

Analysing text data using content analysis involves converting qualitative data in the form of text, including the content and relationships of certain words, categories, and concepts, into numerical forms to facilitate descriptions [33]. In this process, we adhered to the following steps:

#### Defining the student groups

The first step was to clearly define the two student groups being compared. The data were divided into two student groups on the basis of age inclusion criteria: “Group A: Participants aged 19–25 (mean age 20.7 years)” and “Group B: Participants aged 26–47 (mean age 34.3 years)”. Age was chosen as the inclusion criterion to explore potential age-related similarities and differences in the data. Comparing student groups provides a comprehensive understanding of the unique needs, challenges, and strengths of each group [35] – i.e., the contextual demands.

#### Data preparation

Each of the students’ written submissions was read multiple times by three of the authors (MR, HNB, CW) to ensure an understanding of the data [33].

#### Coding of the data

The coding process began with the first author (MR) initially defining the codes based on the data. The codes represent specific ideas, concepts, or features within the data [33]. Inductive coding involves researchers generating codes directly from the data without preunderstanding them, which allows for the discovery of new insights and patterns [41]. The second (HNB) and third (CW) authors then reviewed and validated the predefined codes and also carefully assessed the text for the potential emergence of additional codes. However, no further codes were identified, which confirmed the comprehensiveness of the initial coding framework. This collaborative approach ensured the reliability of the initial coding. During this process, irrelevant information that did not answer the aim was excluded to instead focus on the specific contextual demands that nursing students experienced as challenging their SDL. This step was crucial for distilling the data down to the most pertinent details, thereby providing a clear and focused basis for analysis.

The coding process involved breaking down the text into manageable segments and assigning codes to these segments on the basis of their content. Each meaningful piece of the text was assigned one or more codes that represented the aim of the study. A coding scheme was developed (Supplementary File 1) on the basis of the aim of the study. Once the initial coding was completed, we reviewed the codes to ensure consistency and relevance. This coding process was performed manually.

#### Grouping codes into categories

We grouped related codes into categories. Categories are higher-level groupings that encompass multiple related codes [33, 35]. This step involved merging similar codes and eliminating redundant ones. This involved identifying connections and relationships between codes, which was appropriate for organizing the data into broader categories.

#### Quantifying written submissions

The written submissions containing each category were counted and percentage calculated separately for each student group, reflecting the differences of submissions that included each category across the dataset. This approach provided a clear overview, and helped to identify patterns and trends within the data [33, 34].

#### Comparative analysis

We compared the student groups based on the number of submissions that included the categories. A comparative analysis highlights similarities and differences, and provides insights into patterns, relationships, and context-specific variations [35]. This involved analysing the similarities and differences influencing SDL in the data across two student groups in nursing education.

### Rigour

Instead of evaluating the quality of the written submissions, we concentrated on scrutinizing the specific contextual demands that students encountered during their SDL of biosciences (anatomy, physiology, and biochemistry). In our analysis, we approached all the students’ self-reported written submissions with impartiality and respect, and maintained an unbiased stance throughout the process. To maintain objectivity and ensure a fair and balanced representation of the data within the student submissions, we followed a systematic process [35]. This involved carefully reviewing, coding, and categorizing the information to identify relevant patterns. The authors (MR, HNB, CW) further counted the number of submissions containing each category, which were then expressed as percentages. By doing so, we aimed to provide an accurate and comprehensive overview of the challenges that impacted SDL, comparing the dynamics between two distinct student groups.

### Ethical considerations

Our study was conducted with a strong commitment to ethical standards. Several steps were taken to ensure ethical integrity:

The study obtained ethics approval from the Norwegian Agency for Shared Services in Education and Research (project no. 815027), and the participating university granted approval for this study prior to data collection. In accordance with national regulations, approval from a medical ethics committee was not required, as the study was non-invasive and did not involve sensitive personal information or direct patient interaction. Approval to recruit students was granted by the Head of the Faculty of Health and Social Sciences at the participating university.

The study followed the ethical principles outlined in the Declaration of Helsinki [42]. Participants were fully informed about the aim of the study, the nature of the written submissions, and how their short written text narratives would be used. They provided written consent, acknowledging their voluntary participation and understanding of the study. To protect participants’ privacy, all identifying information was removed from the written submissions, and each document was assigned a unique code to ensure anonymity. Additionally, details about the educational setting and student characteristics have not been disclosed in the methods or results sections of the study. The data were stored securely to prevent unauthorized access.

The content of the written submissions was handled with respect and sensitivity, ensuring that participants’ written narratives were used solely for research purposes and not disclosed in any manner that could identify them. Throughout the analysis process, efforts were made to accurately represent the participants’ contextual demand challenges of SDL without any misinterpretation or distortion of their words.

## Results

Five contextual categories emerged during the analyses, which reflected contextual demands that contributed to the students' learning challenges and appropriate SDL of biosciences: ‘work-related conditions’, ‘social-related conditions’, ‘conditions related to physical activity’, ‘conditions related to social media usage’, and ‘long commute between home and the academic institution’ (Table 1).

**Table 1:** Overview of the number of written submissions containing SDL categories

Categories	Group A: number of submissions	Group B: number of submissions
Work-related conditions	45 of 69 (65%)	12 of 29 (41%)
Social-related conditions	43 of 69 (62%)	23 of 29 (79%)
Conditions related to physical activity	16 of 69 (23%)	1 of 29 (3%)
Conditions related to social media usage	9 of 69 (13%)	1 of 29 (3%)
Long commute between home and the academic institution	7 of 69 (10%)	3 of 29 (10%)

### Work-related conditions

The participants in both groups experienced a heavy workload alongside their nursing studies (Table 1). The participants in the younger student group experienced greater challenges than those in the mature student group in terms of balancing work and SDL, and they expressed a need to reduce their working hours to have more time and energy for their studies. For these younger participants, work was perceived as a stress factor when it clashed with assignment deadlines and exam preparations. One young participant provided an example of work-related conditions that contributed to SDL challenges in this quote:

*I need to work as a permanent employee and on-call substitute in addition to my studies to cover living expenses. This significantly reduces the time available for SDL*,* often preventing me from focusing on schoolwork. It frequently creates a feeling of stress when workdays coincide with important deadlines or exam preparations. (Participant 63*,* Group A)*

Participants in both student groups reported that the need to work came at the expense of their studies, resulting in less time for the SDL of biosciences:

*After a night shift*,* I go home and sleep for most of the day*,* which uses up a lot of time that I could have spent on schoolwork. (Participant 6*,* Group A)*

*I have a permanent job*,* and I cannot study the curriculum while I am at work. (Participant 8*,* Group A)*

*I have regular shifts that sometimes prevent me from preparing for lectures; I cannot manage it while I am at work. (Participant 32*,* Group A)*

*My study time is reduced due to work. (Participant 12, group B)*

*Work and studies are difficult to combine. I often become very tired and exhausted when my mind is in constant work mode. (Participant 15*,* Group B)*

There were economic challenges that made it necessary for participants in both groups to work alongside their studies. For many participants, it was essential to work a certain number of hours per week to ensure a sufficient income:

*Owing to financial challenges*,* I often have to work and use study days for work. (Participant 49*,* Group A)*

*Combining a 50% job and 100% school can sometimes be demanding. I have to plan week by week*,* and no two weeks are the same in terms of both school and work. If I had the opportunity to work less*,* I would*,* but financial considerations play a significant role in making everyday life feasible. (Participant 5*,* Group B)*

Additionally, there were participants in both groups who found it challenging to decline extra shifts outside of their scheduled rota, as they felt it would place a burden on the department if they did not help cover unfilled shifts:

*It is difficult to say no to extra work when there are many people at work on sick leave. (Participant 57*,* Group A)*

*The downside of having a job for me is that I find it hard to say no to extra shifts. This can lead to me working more weekends than planned. (Participant 29*,* Group B)*

In both student groups, the participants found it challenging to spend time on SDL on their working days. After a long workday, the participants were tired and exhausted, which was not conducive to working on their studies:

*After work*,* it can sometimes be difficult to maintain a full focus on studying*,* as one can be tired after a long workday. (Participant 2*,* Group A)*

*When I work*,* it is easy to become tired and lose motivation to study after the workday. (Participant 24, group B)*

### Social-related conditions

Many participants in both student groups described social conditions as a factor that influenced their likelihood of having SDL of biosciences (Table 1). They faced challenges in balancing their SDL and social relationships, as interactions with family, friends, and partners often interfered with their study of biosciences. The younger participants (Group A) primarily described social relationships as including adult relationships, with few students in this group having caregiving responsibilities for children (4%). These challenges are underscored by the following quotes:

*Distractions from friends and/or family can be a challenge*,* especially if they demand my attention when I need self-directed study. I often find it difficult to set clear boundaries or to speak up if I feel they are a disturbance.(Participant 2, group A)*

*I spend as much time as I can with my family*,* as they are the most precious to me. For me*,* nothing matters if my family is not present. Additionally*,* I am a very social person and easily prioritize friends and social events over schoolwork. This means that I often complete schoolwork and assignments on the fly. (Participant 55*,* Group A)*

In the mature student group (Group B), many participants described having parental responsibilities (52%). For participants who were parents (in both groups), it was particularly challenging to find time for SDL, as their children required much attention and care. This was especially true for parents of young and school-aged children. Daily life became hectic with activities that the children needed to participate in, the childrens’ homework, and other family obligations, which made it difficult to spend time on SDL, especially in the evenings after the children had been put to bed, due to fatigue:

*I have children who cry and need comfort at night*,* and this affects my concentration during the day. (Participant 21*,* Group A)*

*I often have to adapt my self-directed studies to the needs of my family. The children require time and attention for activities in the afternoons and homework*,* making it difficult for me to find time to sit down with my studies and complete assignments. (Participant 12*,* Group B)*

In particular, students in the younger student group (Group A) described struggling to maintain a structured study plan due to social obligations but still wished to maintain a social life. Careful planning and prioritization were needed to manage both academic responsibilities and family life:

*It can make it difficult to maintain a structured SDL plan*,* especially when I want to be present and available for those close to me. (Participant 27*,* Group A)*

In the mature student group (Group B), the caregiving responsibility for children made it necessary for participants to plan carefully to balance the time spent on their children and on their SDL:

*I have children and need to plan well to make the time puzzle fit together. (Participant 19*,* Group B)*

In the younger student group (Group A), there were participants who felt guilty for not prioritizing their SDL appropriately:

*It is incredibly enjoyable to be social*,* but I always get a bit of a bad feeling in my stomach afterwards because I feel that I fall behind in my studies. (Participant 41*,* Group A)*

### Conditions related to physical activity

More participants in the younger student group (Group A) than in the mature student group (Group B) described physical activities as time-consuming activities that detracted from their SDL (Table 1). However, the younger students regarded physical activities as an important part of their daily routine. This included both self-initiated activities, such as working out at a gym, and organized sports, where matches were a central component of their physical activity:

*In my free time*,* I participate in sports*,* with two training sessions and up to two matches per week. (Participant 7, group A)*

*I train approximately 4–5 times a week and often have matches on Sundays. (Participant 67*,* Group A)*

Although the participants (in Group A) described the challenges of balancing physical activity and SDL, it emerged that physical activity was important for both physical and mental health. Staying active was crucial for their well-being and study performance:

*I try to fit in four workouts a week at the gym. I cannot simply cut this out*,* because I have an injury that makes it important for me to maintain my muscle strength. (Participant 3*,* Group A)*

*Sports are important for unwinding and clearing my mind. Being active helps me perform better in my schoolwork. (Participant 64*,* Group A)*

In the mature student group (Group B), physical activity did not emerge as a time-consuming demand for students’ SDL, as only one participant briefly mentioned spending time exercising:

*I exercise regularly. (Participant 4, group B)*

#### Conditions related to the use of social media

Several students in the younger student group (Group A) (Table 1) described various technology-based distractions, such as phones, social media, computers, and TV, as challenges for their SDL of the biosciences:

*Social media and phone use are*,* of course*,* factors that can affect self-studying. I often think I will just take a short break on my phone*,* but it quickly turns into an hour because I lose track of time. (Participant 13, group A)*

*Video games constitute a major distraction. They provide quick entertainment and relaxation*,* which can be tempting when I feel mentally tired or need a break. The problem is that breaks with video games often last longer than planned*,* and it becomes challenging to return to my self-studies with full focus. (Participant 27*,* Group A)*

In the mature student group (Group B), only one participant briefly mentioned gaming as a time-consuming factor for their SDL.

#### Long commute between home and the academic institution

In both student groups, a long commute between home and the educational institution was described as a time-consuming factor (Table 1) that could have been better spent on SDL. In the younger student group (Group A), this was identified as a challenge regardless of whether private or public transportation was used, whereas in the mature student group (Group B), time spent on public transportation was not mentioned:

*My commute takes a lot of time away from my self-study time. I do not mind commuting per se*,* but I spend several hours each day just driving. (Participant 29*,* Group B)*

*I commute by train to and from school*,* and I find this more time-consuming than I’d initially thought. It takes me around three hours to get to and from school each day*,* but it can take longer as the train only runs once an hour*,* often at times that do not align well with my classes. (Participant 59*,* Group A)*

## Discussion

This study aimed to increase the understanding of the contextual demands that challenge nursing students’ ability to appropriately engage in the SDL of biosciences in nursing education. Focusing on SDL is crucial, as most students, including novice students, appear to demonstrate self-directed learning readiness [32] but often lack the knowledge and support needed to engage effectively with this learning strategy [19]. The study addresses the demand within the literature for research knowledge on the learning and mastery of biosciences [1, 7, 30, 32]. In the literature review by Royse et al. [30], which included 78 studies, the authors concluded that such a research focus could not only benefit student learning but also contribute to the overall success and reputation of the educational institution. The present study identified five categories of contextual demands that influence nursing students’ ability to effectively engage in SDL of biosciences. These results have the potential to enhance the quality of education in the biosciences for nursing students.

The study results reveal that a considerable number of participants worked alongside their nursing education, primarily due to financial necessity. These results are consistent with findings in previous studies that reported that nursing students face financial challenges while studying full time [21, 43, 44]. For the nursing students in the study by Salamonson et al. [44], working paid hours each week while studying full-time was a financial necessity rather than a choice, as was also found in our study. In our study, we found that younger participants were more likely than mature participants to have a part-time job to finance substantial living expenses such as accommodation and daily costs. Younger students may lack the financial stability of their mature counterparts, which could explain why fewer mature participants in the present study worked alongside their nursing studies. However, participants in both student groups reported that their part-time job contributed to a sense of a heavy workload. Given that nursing educational experiences are perceived by students as demanding and time-consuming [44], important questions are raised about the financial necessity that drives students to seek employment while studying. This was particularly relevant for the students in the study by Ataro et al. [45], which primarily consisted of younger students aged 18–24 years, who reported that many students were immature and faced learning challenges. This might explain why younger participants in the present study struggled with SDL, particularly when faced with a heavy workload. Mature students might have better developed time management skills and a clearer understanding of their academic and professional goals, thereby allowing them to prioritize their studies more effectively [19, 36]. However, data collection for both the present study and that by Ataro et al. [45] took place after the COVID-19 pandemic. In a study comparing pre- and post-pandemic student experiences, Buizza et al. [19] found that younger students in the post-pandemic period were particularly vulnerable and experienced higher levels of mental stress than mature students during the transition to university life. Providing mental health support and resources to assist students in coping with stress, particularly as they adjust to university life, could enhance their well-being and academic performance [46].

In the present study, many of the younger participants prioritized social relationships with others, for both their own well-being and that of others. This emphasis on social interactions aligns with research that suggests that strong social networks can provide emotional support and reduce stress, potentially enhancing academic performance and overall mental health [47]. Although the findings of Zhao et al.’s [47] study are rooted in the context of the COVID-19 pandemic, where isolation and social distancing were major concerns, our study demonstrates that social relationships remain an important focus, even in non-pandemic times. However, social isolation during the COVID-19 pandemic period may account for increased social engagement in the post-pandemic period, as students experienced negative impacts on their social interactions during this time due to a lack of peer interaction [19]. The prioritization of social relationships may also divert time and energy away from academic responsibilities such as SDL, potentially leading to conflicts between social and academic demands [48], which aligns with the results of our study. Guo et al. [48] similarly reported that nursing education demands significant time and effort, as a rigorous curriculum equips future nurses with the comprehensive knowledge needed for the provision of high-quality care. To help students manage these demands, educational institutions should promote peer learning. Although Knutstad et al. [1] found that peer learning did not significantly reduce exam failure rates, collaborative discussions and reflections foster deep learning, which can further enhance students’ SDL efforts [1, 49].

Our results reveal that participants in the mature student group, who often juggled their dual roles as parents and as students, faced a non-negotiable prioritization of their caregiving duties over their SDL. This finding contradicts the results of the study by Ashipala and Natanael [50], which claimed that mothers often experienced a need to prioritize their nursing studies over caring for their children. However, that study was from an African context, where cultural norms and support systems for balancing childcare and nursing education may differ significantly from those in Norway [51]. In Norway, there might be more institutional support and societal acceptance for combining education with parenting responsibilities, whereas in many African cultures, traditional roles and a lack of support structures can make this balance more challenging [50]. Moreover, the participants in the present study were in their initial phase of nursing education, and they might experience the necessity of prioritizing their studies over parenting responsibilities later in their academic journey [52].

The results from the present study indicate a notable difference between younger and mature student groups in terms of the impact of physical activity on SDL. More participants in the younger student group described physical activities as a time-consuming aspect that detracted from their SDL; however, they highlighted the importance of staying active for their overall physical and mental health. Physical activity was deemed crucial not only for maintaining well-being but also for enhancing study performance. These results align with existing research that suggests that regular physical activity can improve cognitive function, reduce stress, and enhance mood, thereby potentially benefiting academic outcomes [53, 54]. In contrast, physical activity did not emerge as a time-consuming demand for participants in the mature student group. Only one participant from this group briefly mentioned spending time exercising, which suggests that physical activities may not have been prioritized or perceived as a major component of their daily routines. This could imply that mature students either have different lifestyle habits or have developed more efficient strategies for integrating physical activity with their academic responsibilities.

The results of this study show that participants from the younger student group faced challenges in their SDL of biosciences due to technology-based distractions such as phones, social media, computers, and TV. This is in accordance with previous results, where the ease of access to various forms of entertainment through technology underscored the broader challenge of balancing leisure activities with the demands of SDL [55]. This suggests that the instant gratification and engagement offered by social media can disrupt study routines and academic focus. In contrast, the participants in the mature student group did not report technology-based distractions with the same frequency or intensity as the younger participants, which suggests that they may have developed more effective strategies for managing their time and minimizing distractions. The inherent complexity of SDL, as outlined by Panadero [17], may make the process of SDL challenging. This contrast between younger and mature student groups highlights the potential impact of maturity and established study habits on the ability to navigate technology-induced interruptions. Established study habits, such as setting goals, structuring routines, and prioritizing tasks, help students to stay focused [23] and minimize technological distractions. These skills enable a balance between leveraging technology as a learning tool and avoiding its disruptive effects. However, the use of technology as a learning tool in SDL should not be underestimated, as it motivates students and facilitates learning [12]. The use of technology proves more effective than traditional textbooks, which are often seen as yielding less effective learning outcomes, although textbooks still encourage better SDL [14].

The results of this study revealed that commuting time was an important factor that impacted SDL across both younger and mature student groups. The participants from both groups highlighted the long commute between home and the educational institution as a time-consuming activity that detracted from their available study time. In the study by Molesworth et al. [21], a long commuting time was not only a time commitment but also a financial concern. Educational institutions could play a supportive role by offering resources and strategies to help students make the most of their commuting time – for example, SDL opportunities could be enhanced by providing access to digital learning materials, promoting the use of educational apps, or encouraging the formation of study groups that can utilize travel time for collaborative learning [56].

### Limitations and future research directions

A limitation of the study could be that short written texts were not combined with interviews, which could have provided an opportunity to follow up on the students’ responses. However, many students participated in the study, and they managed to provide sufficiently detailed answers despite a limited word count in the submission; thus, our data provided a solid basis for analysis and study results. This study did not address the inherent factors that may influence nursing students’ SDL, which is another limitation. Additional research is necessary to better understand these intrinsic factors in bioscience education. These factors may include individual characteristics such as motivation, prior knowledge, time management skills, and learning strategies. A potential avenue for further research could involve exploring why some students are able to overcome contextual challenges in self-directed learning whilst others find it more difficult.

## Conclusions

In the present study, nursing students faced challenges in balancing SDL with demanding responsibilities. Both younger and mature student groups dealt with heavy workloads that disrupted their SDL in biosciences. Economic pressures necessitated working alongside studies, which led to fatigue and diminished study productivity. Social obligations further impacted SDL, with mature students juggling parental duties and younger students striving for a structured study plan while maintaining a social life. Physical activity was crucial for younger students’ well-being, although it was time-consuming, whereas mature students did not experience physical activity as a distraction. Similarly, technology-based distractions such as phones and social media were major barriers for younger students, whereas mature students were less affected. In addition to these challenges, lengthy commutes detracted from SDL opportunities in both groups. These results emphasize the need for targeted strategies in nursing education to address these challenges. By recognizing these issues and providing support systems for effective SDL, educational institutions can improve student outcomes in biosciences.

### Implications for nursing education to support self-directed learning

The findings from this study provide valuable insights into the contextual demands that influence nursing students’ ability to effectively engage in SDL of biosciences. The findings highlight several areas where nursing education can be enhanced to better support students in overcoming these challenges. Flexible course schedules should be offered to accommodate students who need to work alongside their studies, allowing them to balance academic and financial demands more effectively. Additionally, integrating discussions on time management and prioritization into the curriculum could help students to develop essential skills for managing competing responsibilities.

To address the challenges posed by technology, workshops or resources on managing technology use and reducing distractions should be provided. At the same time, nursing education should encourage the use of technology for academic purposes, such as digital learning platforms, educational apps, and e-resources, to direct technology use towards appropriate learning outcomes.

Furthermore, SDL skill development should be integrated into the curriculum, focusing on strategies such as goal setting, self-reflection, and progress evaluation. Providing ongoing support to help students build confidence in managing their own learning is also crucial for fostering their autonomy and success in mastering biosciences. The focus should shift towards ensuring that learning is not solely aimed at passing exams but rather at acquiring knowledge that forms the basis for both patient safety and lifelong learning.

## Supplementary Information

Supplementary Material 1.

## Data Availability

To access the data in this study, please contact the corresponding author.
