# Supplementary material for: Exploring contextual demands that challenge nursing students in their self-directed learning of bioscience: a comparative qualitative content analysis
Source: BMC Med Educ. 2026 Feb 21;26:550. doi: 10.1186/s12909-026-08840-5 (PMC13045138; doi:10.1186/s12909-026-08840-5)
Supplement: Supplementary file 1 — Supplementary Material 1. [file 12909_2026_8840_MOESM1_ESM.pdf]

Supplementary file 1 – the coding scheme

The coding scheme with explanations of codes

Codes	Explanations
- Long working hours<br>- Part-time jobs<br>- Workplace stress/issues	Factors related to a participant's employment or professional responsibilities that influence their opportunities for SDL.
- Family responsibilities<br>- Peer pressure or social expectations	Factors involving personal relationships, social obligations, or interactions that influence a participant's opportunities for SDL.
- Participation in sports teams<br>- Participation in fitness routines.	Factors related to physical exercise or participation in sports that influence a participant's opportunities for SDL.
- Excessive time spent on social media<br>- Social media as a source of distraction	The impact of social media platforms on a participant's opportunities for SDL.
- Time spent commuting<br>- Transportation issues	The challenges of commuting long distances between home and the place of study that influence a participant's opportunities for SDL.
